# Meclofenamic acid represses spermatogonial proliferation through modulating m^6^A RNA modification

**DOI:** 10.1186/s40104-019-0361-6

**Published:** 2019-07-11

**Authors:** Tao Huang, Jiayin Guo, Yinghua Lv, Yi Zheng, Tongying Feng, Qiang Gao, Wenxian Zeng

**Affiliations:** Key Laboratory of Animal Genetics, Breeding and Reproduction of Shaanxi Province, College of Animal Science and Technology, Northwest A&F University, Yangling, 712100 Shaanxi China

**Keywords:** Cell cycle, FTO, Meclofenamic acid, N6-methyladenosine, Spermatogonial proliferation

## Abstract

**Background:**

N6-Methyladenosine (m^6^A), the most prevalent modification in mammalian mRNA, plays important roles in numerous biological processes. Several m^6^A associated proteins such as methyltransferase like 3 (METTL3), methyltransferase like 14 (METTL14), α-ketoglutarate-dependent dioxygenase AlkB homolog 5 (ALKBH5) and YTH domain containing 2 (YTHDC2) are involved in the regulation of spermatogenesis and oogenesis. However, the role of the first detected m6A demethylase, fat mass and obesity associate protein (FTO), in germ cells remains elusive. Elucidation of FTO roles in the regulation of germ cell fate will provide novel insights into the mammalian reproduction.

**Methods:**

Mouse GC-1 spg cells were treated with the ester form of meclofenamic acid (MA2) to inhibit the demethylase activity of FTO. The cellular m^6^A and m^6^A_m_ level were analyzed through high performance liquid chromatography combined with tandem mass spectrometry (HPLC/MS-MS). The cell apoptosis was detected via TUNEL and flow cytometry. The cell proliferation was detected through EdU and western blot. The mRNA level of core cyclin dependent kinases (CDKs) was quantified via q-PCR. RNA decay assay were performed to detect RNA stability. Dual fluorescence assay was conducted to study whether MA2 affects the expression of CDK2 dependent on the m^6^A modification at 3’UTR.

**Results:**

MA2 significantly increased the cellular m^6^A level and down-regulated the expression of CDK1, CDK2, CDK6 and CdC25a, resulting in arrest of G1/S transition and decrease of cell proliferation. MA2 downregulated CDK2 mRNA stability. Additionally, mutation of the predicted m^6^A sites in the Cdk2–3’UTR could mitigated the degradation of CDK2 mRNA after MA2 treatment.

**Conclusion:**

MA2 affected CDKs expression through the m^6^A-dependent mRNA degradation pathway, and thus repressed spermatogonial proliferation.

**Electronic supplementary material:**

The online version of this article (10.1186/s40104-019-0361-6) contains supplementary material, which is available to authorized users.

## Background

Over 140 chemical modifications have been discovered in RNA, of which N6-methyladenosine (m^6^A) is the most abundant one that distributes extensively in about 60% of mammalian mRNA. m^6^A mainly occurs at a common RR (m^6^A) CH motif, in the 3’UTR and near the stop codon [1, 2]. m^6^A modification is catalyzed by the methyltransferase complex composed by METTL3, METTL14, Wilms’ tumor 1-associating protein (WTAP), vir like m6A methyltransferase associated protein (KIAA1429), zinc finger CCCH domain-containing protein 13 (ZC3H13), the CCHC zinc finger-containing protein (ZCCHC4) and other undefined proteins [3–5], which are called “m^6^A writers”. Among these writers, METTL3 is the sole catalytic subunit and the others act as adaptors to keep the stable structure and to enhance RNA-substrate recognition. m^6^A can be erased by “m^6^A erasers” FTO and ALKBH5 [6], and be recognized by “m^6^A readers” such as YTH-domain containing proteins YTHDF1, YTHDF2, YTHDF3 and YTHDC2 [7, 8]. As an epitranscriptomic biomarker, m^6^A participates in the regulation of several post-transcriptional events, including translation, RNA stability, alternative splicing and pre-miRNA processing [9–11]. Increasing evidences have demonstrated that m^6^A plays critical roles in many biological processes, such as differentiation of embryonic stem cells [12], cancer development [13, 14], virus infection [15, 16], neurogenesis, oocyte maturation [17, 18] spermatogenesis [19–21], etc.

Spermatogenesis is a complex process that contains proliferation and differentiation of spermatogonia, meiosis of spermatocytes and post-meiotic spermiogenesis of round spermatids, resulting in generating millions of spermatozoa daily in mammalian testes [22]. This process requires accurately, spatially and temporally regulated patterns of gene expression. Investigating the underlying mechanisms of spermatogenesis will provide theoretical basis for development of novel approaches for male infertility therapy and male contraception. Epigenetic factors, such as histone modifications and DNA modifications, have been reported to play important roles in spermatogenesis [23–26]. However, as an epitranscriptomic biomarker, the roles of m^6^A in the regulation of spermatogenesis remain largely unknown [6]. Importantly, depletion of ALKBH5 leads to retarded spermatogenesis, serious apoptosis in pachytene spermatocytes in mice. Moreover, adult male *Mettl3* ^flox/−^ *Vasa*-*Cre* mice were infertile, with no spermatids in the seminiferous tubules [21]. Similarly, depletion of YTHDC2 leads to infertility as well [19]. Meiosis was arrested at zygotene stage in METTL3 or YTHDC2 knockout mice. Furthermore, simultaneously deletion of METTL14 and METTL3 in advanced mouse germ cells resulted in decrease of sperm motility and increase of abnormal sperm ratio, indicating the significance of m^6^A in spermiogenesis [20].

*Fto* is identified as a candidate gene contributing to the obesity and type II diabetes [27, 28]. FTO belongs to the AlkB related non-haem iron and 2-oxoglutarate-dependent oxygenase superfamily [29], which is able to oxidatively demethylate RNA and DNA. The m^6^A demethylase activity of FTO has been demonstrated to be associated with the splicing of RUNX1 translocation partner 1 (RUNX1T1) [28], an adipogenic regulatory factor. Deficiency of FTO results in reduced adipocyte size and body weight [30–32]. Researches on the phenotypes of FTO knockout mice were mainly focused on the fat mass accumulation, body composition, energy expenditure and other obesity relative phenotypes in these studies. On one hand, the demethylation activity of FTO have not been mentioned in these knockout studies. On the other hand, though FTO showed high expression level in testis, however, effects of FTO on spermatogenesis have been overlooked. Given that another m^6^A demethylase ALKBH5 is involved in the regulation of spermatogenesis [6], we speculated that FTO may also regulate the fate of male germ cells.

Recent studies have reported that meclofenamic acid can selectively compete with FTO for binding to nucleic acid *in vitro*. The ethyl ester form of meclofenamic acid (MA2) selectively inhibited FTO activity and elevated cellular m^6^A level in Hela cells [33]. The objective of this study was to elucidate the effects of MA2 on FTO demethylase activity and the fate of male germ cells. In the present study, we confirmed that MA2 specifically inhibited FTO demethylase activity in spermatogonia. We found that MA2 repressed cell proliferation through prolonging G1 stage. We further demonstrated that the 3’UTR m^6^A sites regulated CDK2 expression through directly affecting its mRNA stability. In summary, our findings suggested that MA2 regulates proliferation and cell cycle progression in spermatogonia through the m^6^A demethylase activity.

## Methods

### Cell culture and drug treatment

The mouse spermatogonia cell line GC-1 spg and HEK293T cells were maintained in Dulbecco’s Modified Eagle’s Medium (GE, SH30022) with 10% fetal bovine serum (Gibco, 12484028). Cells were kept in humidified atmosphere, at 37 °C in the presence of 5% CO_2_. MA2 was kindly provided by the Chinese Academy of Sciences Shanghai Institute of Materia Medica. MA2 powder was dissolved with DMSO to 50 mmol/L and storage at − 20 °C. Spermatogonia were grown to 40% confluence. Subsequently, the culture medium was replaced by medium containing different concentration of MA2. After 24 h and 48 h of culture, cells were washed with Phosphate Buffer Saline (PBS, GE, SH30028) and collected for analysis.

### Antibodies

The primary and secondary antibodies were purchased from commercial sources as follows: Mouse anti-FTO (Santa Cruz Biotechnology, sc-271713), Rabbit anti-Bax (CST, 2772), Rabbit anti-Bcl-2 (CST, 2876), Mouse anti-PCNA (Santa Cruz Biotechnology, sc-56), Mouse anti-Ki67 (Santa Cruz Biotechnology, sc-23900), Rabbit anti m^6^A (Synaptic Systems, 202003), Rabbit anti-Actin (Sigma Aldrich, A2547), Mouse anti Tubulin (Santa Cruz Biotechnology, sc-365791). HRP conjugated goat anti-rabbit IgG (CWbio, CW0156) and HRP conjugated goat anti-mouse IgG (CWbio, CW0102S).

### Detection of cell viability

Cell viability was detected using a CCK-8 cell counting kit (Vazyme, A311–01) following the manufacturer’s instructions. In brief, cells were cultured in 96-well plates and treated with 40, 80 and 120 μmol/L of MA2 and the DMSO control. After incubation for 24 or 48 h, the culture medium containing MA2 was replaced by fresh medium. Ten microliters of CCK-8 were added to each well and incubated for 2 h at 37 °C. The OD values were quantified by Microplate Spectrophotometer.

### Apoptosis assay

TUNEL assay was employed to detect apoptosis. Ten thousands of cells were seeded to each well of the 96-well plate and cultured overnight. After treated with 0, 40, 80 and 120 μmol/L of MA2, cells were fixed with 4% paraformaldehyde for 30 min followed by incubating with 0.1% Triton X-100. The fixed cells were labeled with TUNEL BrightGreen (Vazyme, A112–01). The nuclei were counterstained by [Hoechst 33342 (Thermo, 62249)]. TUNEL positive cells were observed and counted under an inverted fluorescence microscope (Olympus, IX71).

### Cell proliferation assay

Cell proliferation was detected using an EdU Apollo 567 In Vitro Imaging Kit (RiboBio, C10310–1). Briefly, the cells were seeded into 96-well plates (1 × 10^4^ cells per well) and cultured overnight. After incubating with 0, 40, 80 and 120 μmol/L MA2 for 48 h, cells were labeled with 10 μmol/L EdU at 37 °C, 5% CO_2_ for 2 h, fixed in 4% paraformaldehyde for 30 min, followed by staining with fluorescent dye. Cell nuclei were counterstained by Hoechest 33342 (Thermo, 62249). EdU positive cells were observed and counted under an inverted fluorescence microscope (Olympus, IX71).

### Flow cytometric analysis

Spermatogonia were treated with 0, 40, 80 and 120 μmol/L of MA2 for 48 h. The Cells were detached with 0.25% (*w*/*v*) trypsin and washed with PBS. Subsequently, cells were kept in 70% cold ethanol at − 20 °C overnight. The cells were incubated with 50 μg/mL of propidium iodide (Sigma Aldrich, 81845) at 37 °C in dark for 30 min, and analyzed with a flow Cytometer (Cyflow Cube). The cell cycle data were analyzed using the software Modfit.

### RNA isolation, cDNA synthesis and quantitative RT-PCR

Total RNA was extracted using Trizol reagent (TAKARA, 9108), followed by Chloroform centrifugation and isopropanol precipitation. CDNA was synthesized using Primescript RT reagent Kit (Roche, 04896866001). Q-PCR was performed using the SYBR Green II PCR Mix (TAKARA) and the fluorescence ration PCR instrument IQ5 (Bio-Rad). The qRT-PCR primers used were listed in Additional file 2: Table S1.

### Western blot assay

Cells were lysed with RIPA lysis buffer (Solarbio Life Sciences, R0020) containing 1% PMSF followed by ultrasonication. Cell lysates were incubated on ice for 30 min, followed by centrifuging at 4 °C for 10 min. The protein concentration of the supernatant was quantified using a BCA Protein Assay Kit (TAKARA, T9300A). The proteins were separated through SDS-PAGE using the electrophoresis apparatus (Bio-Rad). Subsequently, the proteins were transferred to a polyvinylidene fluoride (PVDF) membrane (Millipore, IBFP0785C) using a semi dry transfer instrument (Bio-Rad). The membranes were blocked with 5% non-fat milk at room temperature for 1 h, and incubated with primary antibodies at 4 °C overnight. After overnight incubation, the membranes were washed with PBST and incubated with HRP-conjugated secondary antibodies at room temperature for 1 h, followed by incubation with the Immobilon Western Chemiluminescent HRP Substrate (Millipore) and photographed using the ECL imaging system (Bio-Rad).

### m^6^A/m^6^A_m_ dot-blot

mRNA was purified using PolyATtract® mRNA Isolation Systems (Promega, Z5310) following the manufacturer’s instructions. mRNA was denatured at 95 °C for 5 min and loaded onto a Hybond-N+ membrane (GE HealthCare, RPN303B). After closslinking under ultraviolet radiation, the membrane was blocked with 5% non-fat milk (Bio-Rad) for 1 h, incubated with rabbit anti-m6A polyclone antibody (Synaptic Systems, 202003) at 4 °C overnight. Then the membrane was incubated with HRP-Conjugated goat anti-rabbit IgG (CWbio, CW0156) at room temperature for 2 h. After being incubated with a Immobilon Western Chemiluminescent HRP Substrate (Millipore, WBKLS0500), the immunocomplex was photographed using the ECL imaging system. Finally, the membrane was stained with 0.02% methylene blue to eliminate the difference in mRNA amount.

### Plasmids construction and transfection

cDNA of the cells was synthesized as mentioned above. The DNA fragments of CREBBP-3’UTR and CDK2-3’UTR were amplified via PCR. Mutation of CREBBP-3’UTR and CDK2-3’UTR were generated through the overlapping PCR followed by homologous recombination using a NovoRec recombinase (Novoprotein, NR001). The purified 3’UTR fragments were constructed to pCMV-mCherry-EF1α-GFP vector. For dual-fluorescence protein report assay, plasmids were transfected to HEK-293 T Cells using the TurboFect™ Transfection Reagent (Thermo Fisher, R0534) following the manufacturer’s instructions. After 48 h, the fluorescence intensity was detected by a Synergy Muti-Mode Microplate Reader (BioTek). The relative fluorescence intensity (RFI) was calculated as follows: RFI = FI (red) / FI (green).

### Quantitative analysis of RNA modification levels

The quantitative analysis of m^6^A and m^6^A_m_ level was performed according to previously described [34]. Briefly, total RNA was isolated from cells treated with 0 μmol/L, 40 μmol/L, 80 μmol/L and 120 μmol/L MA2, respectively. mRNA was purified using the PolyATtract® mRNA Isolation Systems (Promega, Z5310) following the manufacturer’s instructions. Three hundred micrograms of mRNA was further treated with 3 μL RppH (NEB) at 37 °C for 2 h. After the removel of cap, mRNA was treated with nuclease P1 (Sigma, N8630) at 42 °C for 2 h. Afterwards, the ribonucleotides were treated with alkaline phosphatase (Sigma, P5931) at 37 °C to remove the phosphate group. The amount of adenosine (A), m^6^A and m^6^A_m_ was measured according to HPLC-tandem mass spectrometry. The ratio of m^6^A to A and m^6^A_m_ to A were calculated based on the calibrated concentrations.

### RNA-decay assay

Cells were treated with 0, 40 and 80 μmol/L MA2 for 24 h. Then the culture medium were added 5 μg/mL actinomycin D and incubated for 0 h, 3 h and 6 h, respectively. Cells were harvested and subjected to RNA extraction. Real-time quantitative PCR were used to analyze the mRNA level of the target genes in each group.

### FTO-RIP-PCR

For FTO-RIP, the full CDS of *Fto* gene was amplified by high fidelity PCR. The *Flag* sequence was inserted to the 5′ end of *Fto*-CDS. The Flag-Fto sequence was inserted into the CD513B-CMV plasmid. Flag-GFP was used as negative control. Anti-FLAG-based RIP was performed with modifications according to [35]. Breifly, CD513B-Flag-FTO and CD513B-Flag-GFP were transfected to the GC-1 cells, respectively. After 24 h culture, cells were collected and lysed by the lysis buffer, while 1/10 volume of cell lysates were preserved as input. Protein A beads were coated with 10 μg rabbit anti-FLAG antibody. Cell lysates and beads were mixed and rotated at 4 °C overnight. After washed for five times, beads and input lysates were incubated with proteinase K at 55 °C for 30 min followed by centrifugation. IP RNA was extracted from the supernatant using TRIZOL followed by chloroform/ isopropanol centrifugation. RT-PCR was performed to normalize the mRNA level of target genes.

### Statistical analysis

All data were collected from at least three independent experiments. Data were analyzed using two-tailed student’s *t-*test or One-way ANOVA followed by a Duncan’s multiple range test (SPSS 22 for windows), with *P* < 0.05 Considered statistically significant.

## Results

### MA2 elevates cellular m^6^A and m^6^A_m_ level and decreases cell viability

**Fig. 1:**
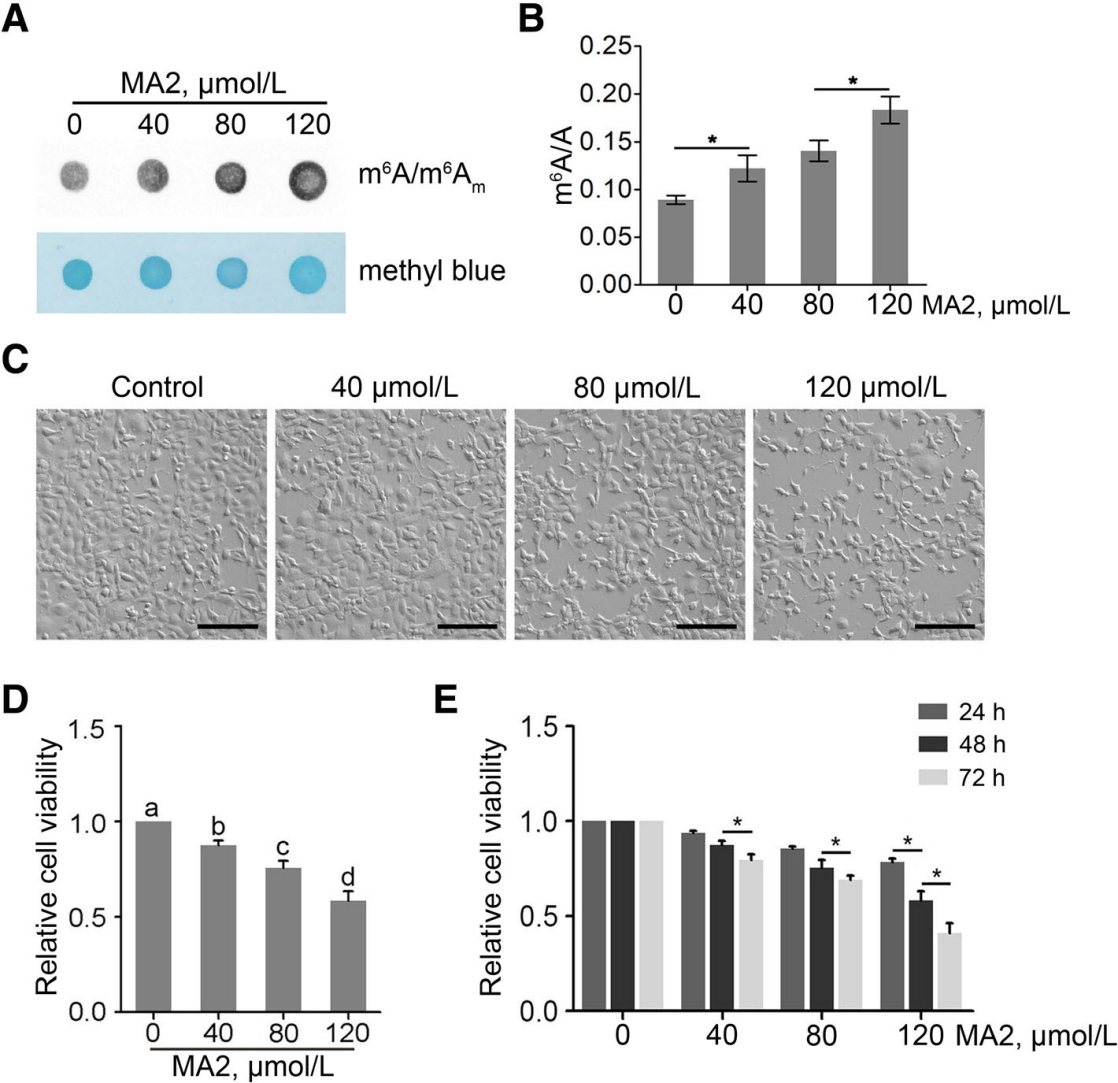
Phenotypes of spermatogonia treated with MA2. **a** Effect of MA2 on the cellular m^6^A level. GC-1 cells were treated with 0, 40, 80 and 120 μmol/L of MA2 for 48 h. Cellular m^6^A level was analyzed via m^6^A dot blot. **b** Quantification of cellular m^6^A level through HPLC/MS-MS. Data were presented as the mean ± SEM, *n* = 3. *P < 0.05. **c** Images of the GC-1 spg cells. Cells were treated with 40, 80 and 120 μmol/L of MA2 or the carrier DMSO for 48 h in triplicates. The morphology of cells was photographed. Bar = 50 μm. **d** Dose-dependent effect of MA2 on cell viability. GC-1 cells were treated with MA2 for 48 h. Different lower-case letters denoted significant differences (*P* < 0.05). **e** Time-dependent effect of MA2 on cell viability. GC-1 cells were treated with 0, 40, 80 and 120 μmol/L MA2 in triplicates. Cell viability was detected via CCK-8 assay. Data were presented as the mean ± SEM, *n* = 3. *P < 0.05

To investigate whether MA2 affect the m^6^A level in spermatogonia, we treated the cells with 0, 40, 80 and 120 μmol/L of MA2 for 48 h, respectively. The m^6^A/m^6^A_m_ level was detected by dot blot and HPLC/MS-MS. As shown in Fig. 1a and b, MA2 enhanced cellular m^6^A level in a dose-dependent manner. Additionally, MA2 did not affect the m^6^A_m_ level of polyA RNA. However, MA2 increased the m^6^A_m_ level of small nuclear RNA in a dose- dependent manner (Additional file 1: Figure S1). We then detected the cell morphology under the microscopy. As shown in Fig. 1c, MA2 did not affect cell morphology, but led to a decrease in cell density. We next detected the cell viability via CCK-8 assay, and found that the cell viability significantly decreased in both dose and time dependent manner (Fig. 1c and d). Thus, MA2 efficiently elevated the m^6^A and m^6^A_m_ level and significantly reduced cell viability of spermatogonia.

### Effects of MA2 on spermatogonial apoptosis and proliferation

**Fig. 2:**
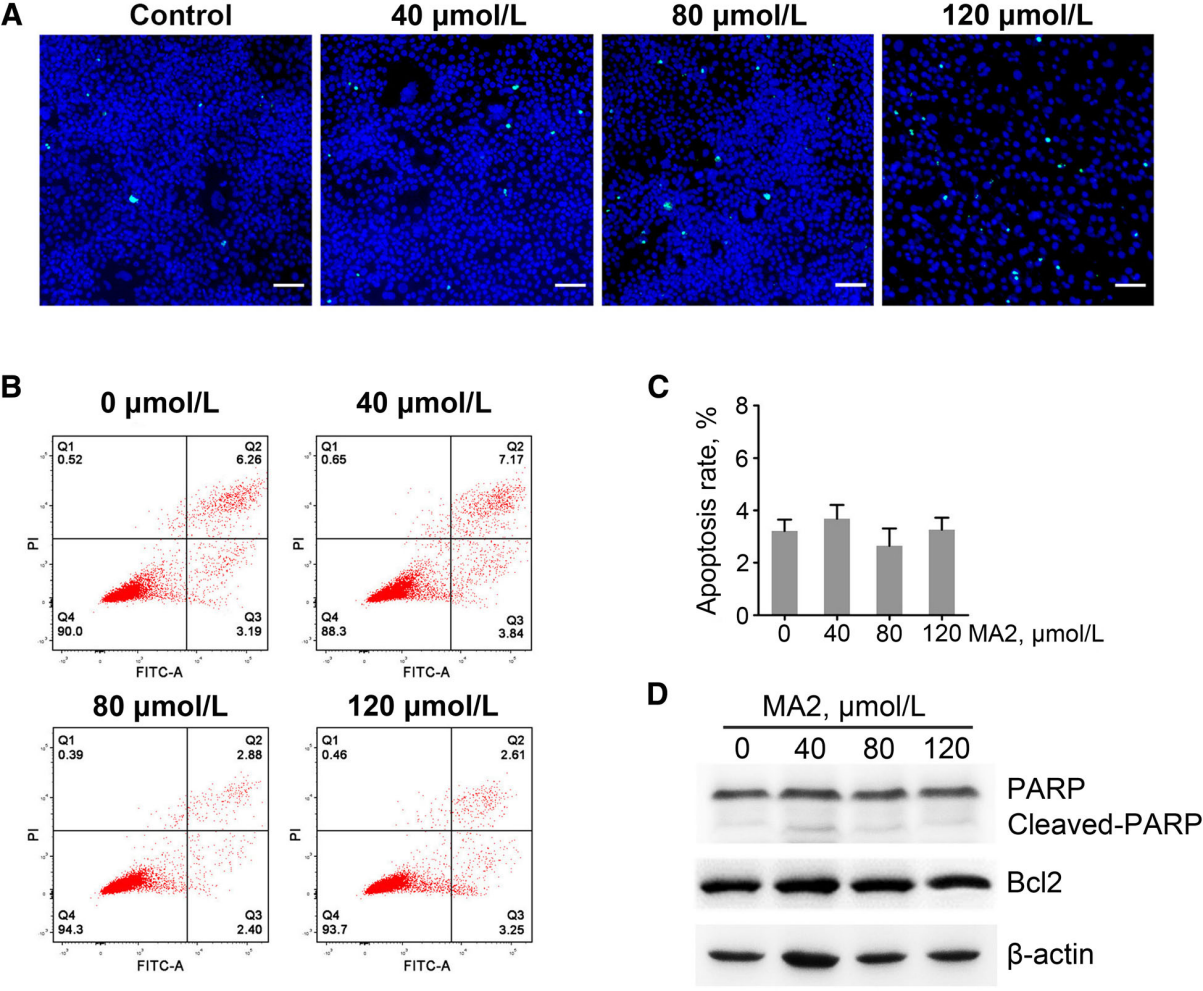
Effect of MA2 on cell apoptosis. **a** GC-1 cells were treated with 0, 40, 80 and 120 μmol/L MA2 for 48 h. TUNEL assay was used to analyze apoptosis. TUNEL positive cells were indicated by green fluorescence. Cell nucleic were stained by DAPI (blue). Bar = 50 μm. **b** GC-1 cells were double-stained with PI/Annexin V and analyzed by flow cytometry. Q4 represents non-apoptotic cells, Q3 represents apoptotic cells, and Q2 represents dead cells. **c** Apoptotic rates of all groups were showed in column diagram. Data were represented by mean ± SEM, (*n* = 3 independent replicates).**d** Effects of MA2 on the abundance of PARP, cleaved PARP and Bcl2. β-Actin was used as the loading control

To investigate whether the treatment of MA2 led to cell apoptosis, we treated the GC-1 cells with 0, 40, 80 and 120 μmol/L of MA2 for 48 h. TUNEL assay showed that positive rate was not different between the MA2 treatments and the control (Fig. 2a and c), indicating that MA2 did not cause apoptosis. To further verify the phenotype, we performed the PI/Annexin V staining followed by flow cytometry. As shown in Fig. 2b, apoptosis rates (quantitative value in quadrant 3) in all treated groups were not different, which was accordant to the TUNEL assay result.

We further detected the expression of apoptosis relative protein PARP and anti-apoptosis protein Bcl2. As shown in Fig. 2d, the level of PARP in treated groups was comparable to the control, and expression of the cleaved-PARP in each group was hard to observe. Additionally, the level of anti-apoptosis protein Bcl2 was similar in all the groups. Taken together, these results suggest that MA2 did not induce cell apoptosis.

**Fig. 3:**
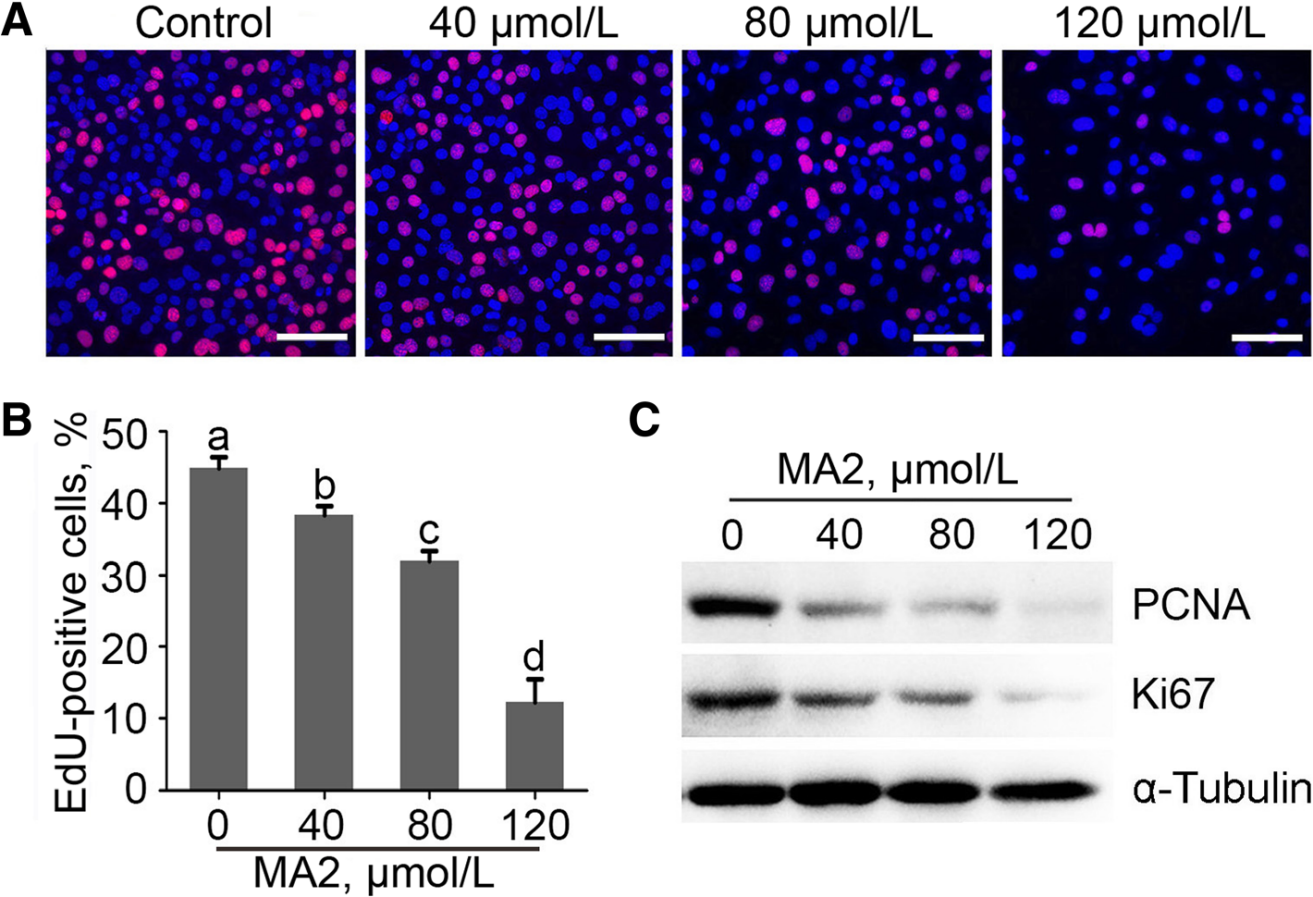
Effect of MA2 on cell proliferation. **a** GC-1 cells were treated with 0, 40, 80 and 120 μmol/L of MA2 for 48 h. EdU assay was performed to analyze cell proliferation. EdU positive cells were indicated by red fluorescence. Nuclei were stained by DAPI (blue), bar = 50 μm. **b** EdU positive rates were showed in column diagram. Data were represented by the mean ± SEM, *n *= 3. Different lower-case letters denoted significant differences (*P* < 0.05). **c** Effects of MA2 on expression of the proliferation markers. GC-1 cells were treated with different concentration of MA2 for 48 h. Expression of PCNA and Ki67 were detected by western blot. α-Tubulin was used as the loading control

To detect cell proliferation, we performed EdU assay, which measures the rate of DNA replication and labels S-phase cells [36]. Cells were treated with 0, 40, 80 and 120 μmol/L of MA2 for 48 h. EdU assay showed that MA2 caused an intensive decrease of EdU positive cells (Fig. 3a and b). Meanwhile, the expression of proliferation markers PCNA and Ki67, that are expressed in active phases of cell cycle [37, 38], drastically decreased in the MA2 treatments (Fig. 3c), indicating that cell proliferation was dramatically repressed by MA2. These observations suggested that MA2 repressed cell proliferation.

### MA2 arrests cell cycle via down-regulating mRNA level of CDKs

**Fig. 4:**
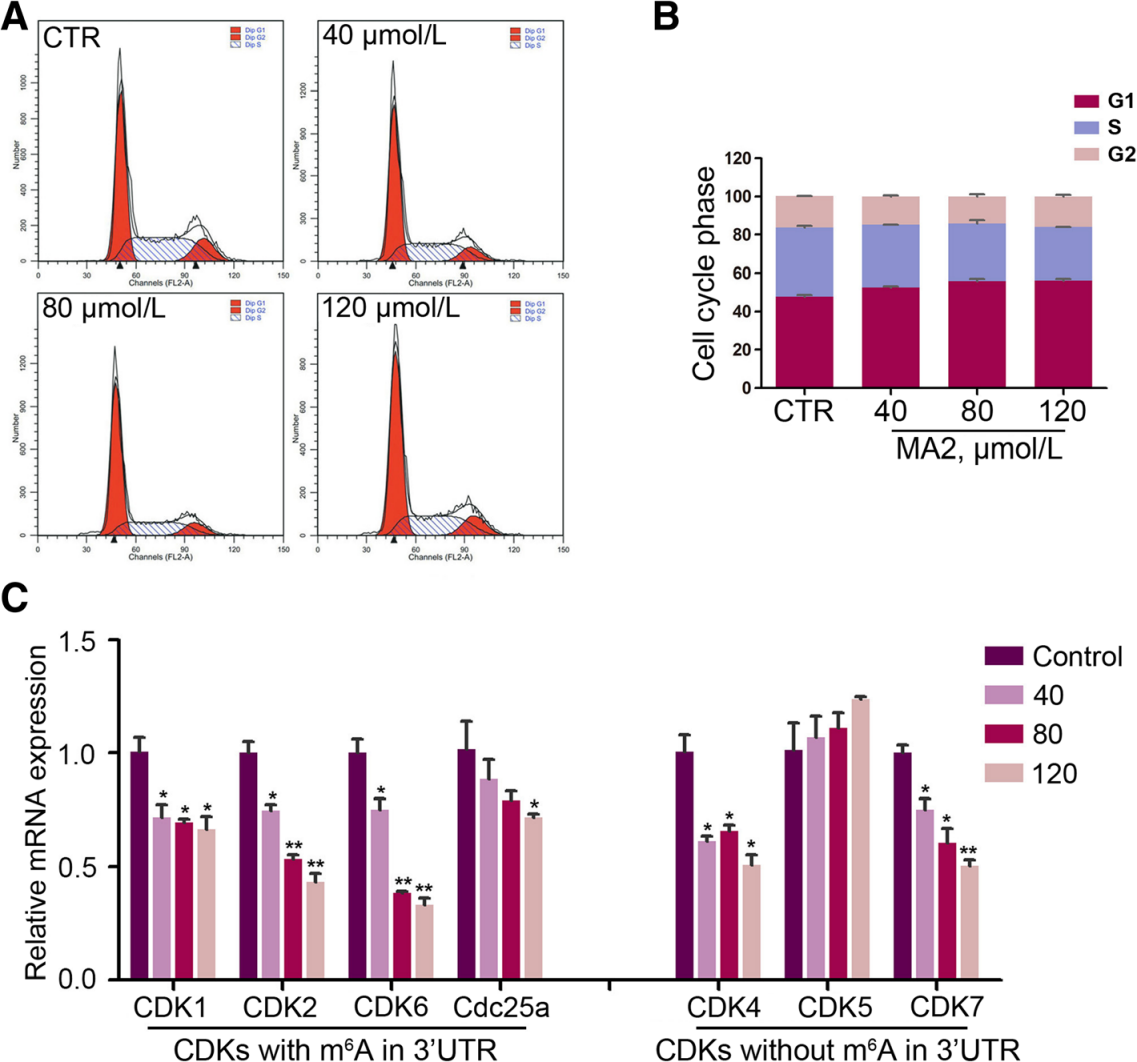
Effects of MA2 on progression of cell cycle. **a** Cells were treated with 40, 80 and 120 μmol/L of MA2 or the carrier DMSO for 48 h. Subsequently, the cells were harvested and stained with propidium iodide and analyzed using a flow cytometry. (A) Cell cycle phase was analyzed by ModFit. **b** Quantification of percentage of cells in each cycle phase. Data were represented by the mean ± SEM, *n* = 3. **c** Relative mRNA expression of CDKs involved in G1/S transition. *P < 0.05, **P < 0.01. Data were represented by mean ± SEM, *n* = 3

Previous studies showed that m^6^A was involved in the regulation of cell cycle during oocyte maturation in *Xenopus laevis* oocytes [17]. To determine whether MA2 affects the cell cycle transition, we analyzed the cell cycle through flow cytometry. Interestingly, MA2 treatment led to a significant increase of cell numbers in G0/G1 phase and a dramatic decrease in S and G2/M phase (Fig. 4a and b), indicating that MA2 arrested the G1/S transition.

As the cell cycle progression is elaborately regulated by the cyclin-dependent kinases (CDKs), to investigate whether MA2 affect CDK expression, we searched the online database of RNA methylation (http://rna.sysu.edu.cn/rmbase/) and refered the RNA methylation information of the core CDKs that are involved in G1/S transition. As shown in Additional file 2: Table S2, CDK1, CDK2, CDK6 and CdC25a contained m^6^A modified sites in the 3’UTR, while CDK4, CDK5 and CDK7 did not. Subsequently, the mRNA level of the seven CDKs was measured through q-PCR. Interestingly, the mRNA level of CDK1, 2, 6 and CdC25a was significantly down-regulated by MA2. However, the mRNA level of CDK4 and CDK7 was also down-regulated by MA2, while expression of CDK5 showed no difference (Fig. 4c). Together, these results suggested that MA2 affected the expression of CDKs involved in G1/S transition in spermatogonia.

### CDK2 mRNA level dependent on 3’UTR m^6^A modification

**Fig. 5:**
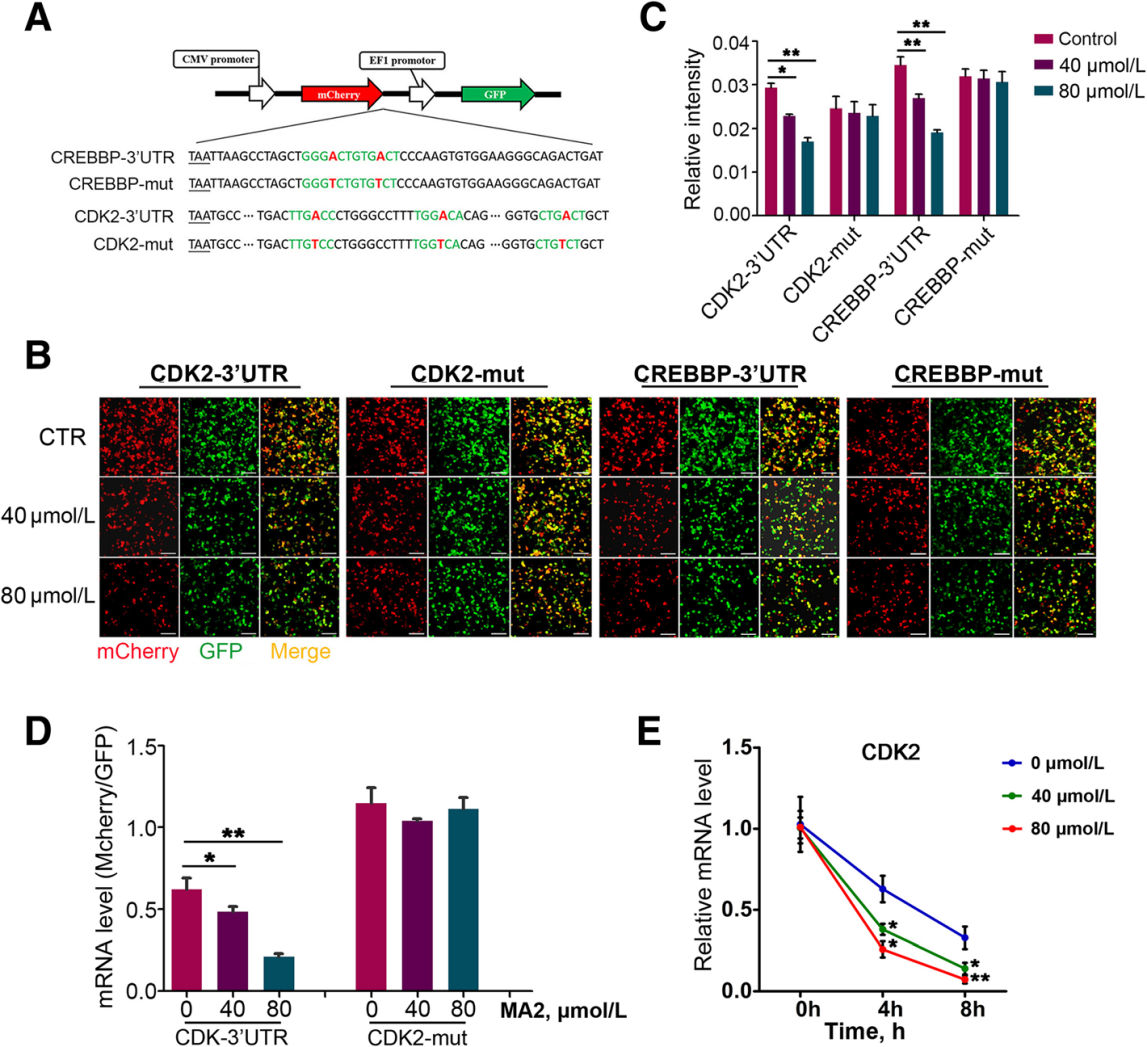
MA2 downregulated CDK2 expression and cell viability through the 3’UTR-m^6^A mediated mRNA degradation. **a** Schematic diagram of plasmids used in the 3’UTR fluorescence reporter assay. Letter A and T (red) represented the mutation of predicted m^6^A to T. **b** Effects of CDK2-3’UTR and CDK2-mut on the expression of mCherry. Cells were treated with 40 and 80 μmol/L of MA2, and simultaneously transfected with CDK2-3’UTR and CDK2-mut plasmids. After 48 h, the cells were photographed under the fluorescence microscope. Bar = 50 μm. **c** Relative fluorescence intensity was showed in column diagram. Data were represented by the mean ± SEM, *n* = 3. *P < 0.05, **P < 0.01. **d** Relative mRNA level of mCherry to GFP in each group. *n* = 3 independent duplications, *P < 0.05, **P < 0.01. **e** Cells were treated with 0, 40 and 80 μmol/L MA2 for 24 h. After MA2 treatment, cells were treated with 5 μmol/L actinomycin D for 4 h and 8 h, respectively. Remained RNA level was quantified by RT-PCR. Data were represented by the mean ± SEM, *n* = 3. *P < 0.05, **P < 0.01

Previous studies have demonstrated that the 3’UTR m^6^A modification can accelerate mRNA degradation [7]. Considering that the CDK2 mRNA harbored three m^6^A sites in the 3’UTR, we assumed that MA2 down-regulated the CDK2 mRNA level through accelerating mRNA degradation. To verify the hypothesis, we performed a dual fluorescence assay. Three predicted methylated adenines in the 3’UTR of CDK2 were mutated to thymine (Fig. 5a). Considering that the m^6^A sites within the CREBBP-3’UTR have been reported to downregulated the stability of CREBBP mRNA [7], we employed the CREBBP-3’UTR as positive control. Briefly, CDK2-3’UTR and CREBBP-3’UTR were respectively constructed to the downstream of a coding sequence (CDS) that encoded a red fluorescence protein (RFP). The relative fluorescence intensity (RFI, red to green) indicated the expression of CDK2 and CREBBP. Interestingly, MA2 caused a significant decrease of the RFI in the cells that were transfected with CDK2-3’UTR. Whereas, in CDK2-mut cells, it was similar between the control and MA2 treated groups. In addition, RFI of the CREBBP-3’UTR group and CREBBP-mut group was consistent with that in CDK2 (Fig. 5b and c). We further quantified the mRNA level through RT-PCR. As shown in Fig. 5d, the tendency of mRNA level was similar to that of the RFI, indicating that MA2 reduced the mRNA level of CDK2 through elevating the 3’UTR m^6^A modifications.

Previous studies have demonstrated the important role of m^6^A in mRNA degradation [7]. Therefore, we speculated that MA2 down-regulated CDK2 expression through suppressing mRNA stability. To this end, we performed the RNA decay experiment. Cells were treated with 0, 40 and 80 μmol/L of MA2 for 24 h, then subjected to 5 μmol/L of actomycin D to abolish the synthesis of new transcripts. After 4 h and 8 h treatment, the remained mRNA level of *Cdk2* was quantified by RT-PCR. As shown in Fig. 5e, the *Cdk2* mRNA level in the MA2 treated cells was significantly lower than the control, indicating that MA2 repressed CDK2 stability.

**Fig. 6:**
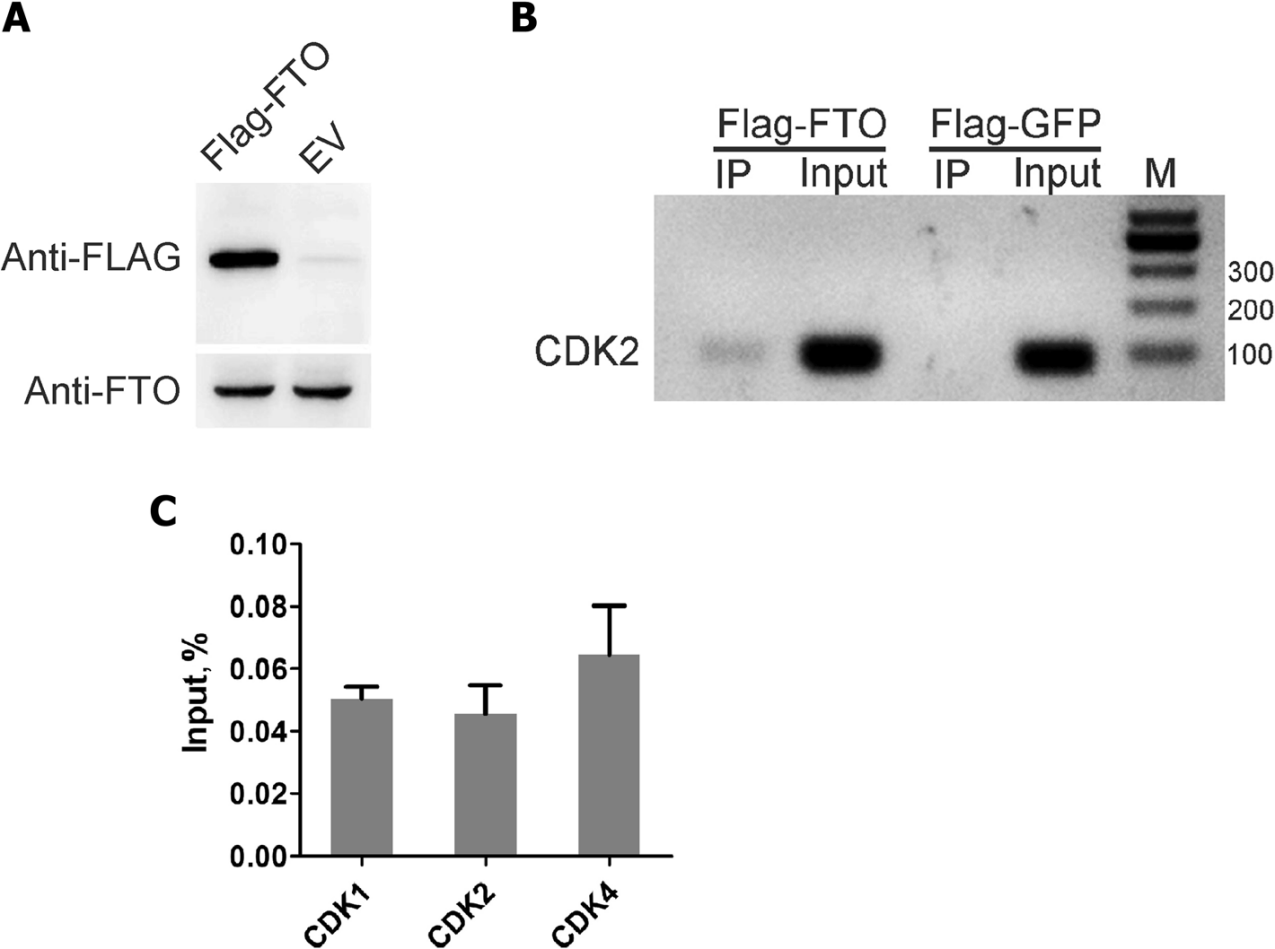
Anti-FLAG-based RNA immunoprecipitation coupled to RT-qPCR. **a** Validation of Flag tagged FTO expression by Western blot. β-actin was used as the loading control. **b** Electrophoretogram of the RT-PCR products using immunoprecipitated RNA and *Cdk2* primer. **c** RNA IP confirmation of FTO binding to RNAs identifed by RT-qPCR

To further validate the interaction between FTO and CDKs mRNA, we performed the Flag based FTO-RIP. CD513B-Flag-FTO and CD513B-Flag-GFP were transfected to GC-1 cells, respectively. Cells were collected and subjected to RIP using anti-FLAG antibody. We found that mRNA of CDK1, CDK2 and CDK4 could be detected in the IP RNA, while CDK5, CDK6 and CDK7 could not (Fig. 6b and c), indicating that FTO directly interacted with CDK1, CDK2 and CDK4.

Together, these results suggested that MA2 regulated CDK2 expression, at least partially, through the m6A/mRNA degradation pathway.

## Discussion

In the present study, we found that MA2, by inhibiting FTO demethylase activity, arrested G1/S transition and suppressed spermatogonial proliferation through downregulating CDK expression. We further demonstrated that MA2 accelerated the degradation of CDK2 mRNA, thus led to down-regulation of CDK2 expression through elevating the 3’UTR m^6^A level.

Growing evidences have indicated the significance of m^6^A in regulation of cell proliferation [39–42]. Deletion of METTL3 disrupted homeostatic proliferation of mouse naïve T cells [39]. Similarly, silencing ALKBH5 in glioblastoma stem cells suppressed cell proliferation via enhancing FOXM1 expression [41]. Moreover, FTO promotes proliferation of AML cells through targeting of the ASB2 and RARA in acute myeloid leukemia cells [42]. In addition, FTO regulated proliferation and differentiation of adult neural stem cells through modulating the BDNF/Akt signaling pathway [40]. Here, we revealed the regulatory role of m^6^A demethylase in the proliferation of spermatogonia.

MA, as a member of the non-steroidal anti-inflammatory drug, is well known for its inhibitory role in prostaglandins synthesis [43]. MA can inhibit the activity of cytochrome c oxidase subunit 2 [44], which promotes proliferation of various cancer cells. As such, MA possesses the therapeutic potential in different types of cancer patients [45, 46]. In the present study, we showed that MA2, the ester form of MA, elevated cellular m^6^A level in spermatogonia, which was accordant to that in Hela cells [33]. We found that MA2 repressed cell viability through inhibiting the demethylase activity of FTO, providing new insights into the mechanisms of MA suppressing cancer cell proliferation.

The transition from G1 to S stage is essential for cell proliferation. In the present study, we showed that inhibiting the demethylase activity of FTO down-regulated the CDKs that were associated with G1/S transition. Among the detected genes, CDKs 1, 2, 6 and CdC25a possessed m^6^A sites in the 3’UTR. Previous studies have adequately demonstrated that increased m^6^A modifications in the 3’UTR will reduce mRNA stability through interacting with the m^6^A reader YTHDF2, YTHDF3 or YTHDC2 [7, 8, 47]. In the present study, we found that MA2 treatment significantly accelerated the degradation of CDK2 mRNA, resulting in downregulation of CDK2 expression and spermatogonial proliferation. In the dual fluorescence assay, we mutated the predicted methylated adenosine in the CDK2 3’UTR. Actually, the mutation of adenosine could potentially also prevent the binding of RNA binding proteins that depend on adenosine, and the contribution of other binding proteins in the phenotypes should not be overlooked.

The mRNA level of several CDKs without m^6^A modifications in the 3’UTR, such as CDK4 and CDK7, was also downregulated by MA2. These results suggested that MA2 regulates the transition of G1/S probably through other pathways. Indeed, previous studies have reported that MA in the inhibited cancer cell proliferation [45, 48]. Sekine et.al reported that combination of simvastatin and MA suppressed the proliferation of prostate cancer cells through inhibition of the expression of aldo-keto reductase family 1 member C3 (AKR1C3). The MA dosage was 50 μmol/L, which was comparable to the dosage of MA2 used in the present study. Considering that MA2 have a stronger ability to pass through the cell membrane, it is obvious that one of the potential off-target proteins in the present study may be AKR1C3. Additionally, partial of MA2 potential targets were listed in Additional file 2: Table S3. The other m^6^A demethylase ALKBH5 has been reported involves in the regulation of spermatogenesis. Deficiency of ALKBH5 induces serious apoptosis in male germ cells in mouse. On one hand, the function of ALKBH5 in spermatogonial proliferation has not been mentioned. On the other hand, the selective activity of MA towards FTO has been shown in recent study. Huang et.al reported that MA could not inhibit the ALKBH5-mediated dm^6^A conversion to adenine in RNA even at high concentrations up to 0.5 mmol/L *in vivo*. Therefore, we suggest that the concentration of MA2 used in our present study have no effect on ALKBH5, and the phenotypes induced by MA2 was not associated with ALKBH5.

Recent studies have reported that m^6^A was not the unique substrate of FTO. FTO can also demethylate at the N6,2′-O-dimethyladenosine (m^6^A_m_). (2) Mauer et al. reported that FTO preferentially demethylated m^6^A_m_ rather than m^6^A, and reduced the stability of m^6^A_m_ mRNAs [49]. Recently, (2) Wei et al. reported that the FTO-mediated demethylation has a greater effect on the transcript levels of mRNAs possessing internal m^6^A than the ones with cap m^6^A_m_ [50]. In the present study, we detected both m^6^A and m^6^A_m_ level of cells treated with MA2, and found that MA2 treatment significantly increased the m^6^A level of mRNA and the m^6^A_m_ level of small nuclear RNA. Since these two types of RNA modifications affect the expression of target genes [51], indicating that MA2 suppressed spermatogonial proliferation probably not only through m^6^A, but also via other pathways such as m^6^A_m_.

Since the CDK2-Cyclin E complex plays important roles in metaphase II (MII) arrest in oocytes [52] and formation of synaptonemal complex in spermatocytes [53], the CDK2 is important for both mitosis and meiosis. Here, we demonstrate that the m^6^A modification in *Cdk2* 3’UTR is related with the CDK2 mRNA stability. This finding is accordant to the recent research on *Xenopus laevis*, which demonstrated that m^6^A was associated with cell cycle and protein translation during oocyte maturation [17]. Recent studies reported that the meiosis was arrested at zygotene stage, with abnormal dissociation of synaptonemal complex when METTL3 or YTHDC2 was depleted in spermatocytes [54, 55]. This phenotype is similar to that of CDK2 deletion [21, 56]. Collectively, we propose that FTO also may play key regulatory roles in meiosis through regulating CDK2 expression. Since the FTO depletion mice exhibits developmental retardation, FTO conditional KO mouse model is needed to further investigate the functions in detail.

## Conclusion

Our findings suggest that MA2 affects male germ cell proliferation through an RNA methylation pathway, thus put forward new insights into the regulatory mechanisms of RNA methylation in spermatogenesis.

## Additional files

Additional file 1: **Figure S1.** Detection of m^6^A_m_ level in polyA RNA and small nuclear RNA. Cells were treated with 0, 40, 80 and 120 μmol/L MA2. Content of m^6^A_m_ and A was detected by LC/MS-MS. Relative m^6^A_m_ level was normalized by m^6^A_m_/A. (A) Relative m^6^Am level of PolyA RNA. Data were represented by the mean ± SEM, *n* = 3. n,s means *P* > 0.05. (B) Relative m^6^A_m_ level of snRNA. Data were represented by the mean ± SEM, *n* = 3. (JPG 648 kb)

Additional file 2: **Table S1.** Oligo information. **Table S2.** m6A modification information of CDKs. **Table S3.** Information of potential MA2 targets. (DOCX 19 kb)

## Abbreviations

ALKBH5: α-ketoglutarate-dependent dioxygenase AlkB homolog 5
CdC25a: Cell division cycle 25A
CDK: Cyclin-dependent kinase
CREBBP: CREB binding protein
FTO: Fat mass and obesity protein
Ki67: Marker of proliferation Ki-67
m^6^A: N6-methyladenosine
MA: Meclofenamic acid
MA2: Ester form of meclofenamic acid
METTL14: Methyltransferase like 14
METTL3: Methyltransferase like 3
PARP1: Poly (ADP-ribose) polymerase family member 1
PCNA: Proliferating cell nuclear antigen
RFI: Relative fluorescent intensity
RUNX1T1: Runt-related transcription factor 1
YTHDC2: YTH domain containing 2
