# Supplementary figures and images for: Meclofenamic acid represses spermatogonial proliferation through modulating m6A RNA modification
Source: J Anim Sci Biotechnol. 2019 Jul 11;10:63. doi: 10.1186/s40104-019-0361-6 (PMC6621992; doi:10.1186/s40104-019-0361-6)

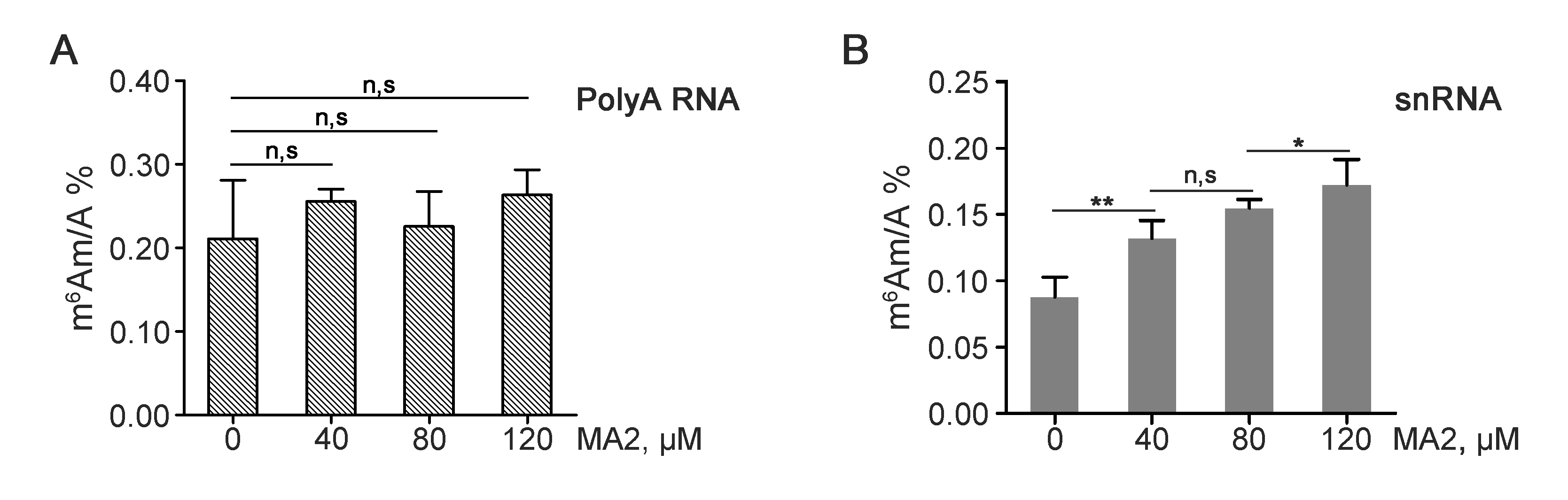

Supplement: Supplementary file 1 — Figure S1. Detection of m6Am level in polyA RNA and small nuclear RNA. Cells were treated with 0, 40, 80 and 120 μmol/L MA2. Content of m6Am and A was detected by LC/MS-MS. Relative m6Am level was normalized by m6Am/A. (A) Relative m6Am level of PolyA RNA. Data were represented by the mean ± SEM, n = 3. n,s means P > 0.05. (B) Relative m6Am level of snRNA. Data were represented by the mean ± SEM, n = 3. (JPG 648 kb) [file 40104_2019_361_MOESM1_ESM.jpg]
