# Supplementary material for: Meclofenamic acid represses spermatogonial proliferation through modulating m6A RNA modification
Source: J Anim Sci Biotechnol. 2019 Jul 11;10:63. doi: 10.1186/s40104-019-0361-6 (PMC6621992; doi:10.1186/s40104-019-0361-6)
Supplement: Supplementary file 2 — Table S1. Oligo information. Table S2. m6A modification information of CDKs. Table S3. Information of potential MA2 targets. (DOCX 19 kb) [file 40104_2019_361_MOESM2_ESM.docx]

**Additional file 2**

**Table S1. Oligo information**

| Oligo name | Sequence (5’ - 3’) |
| --- | --- |
| CDK1-F | CGGTTGACATCTGGAGTATAGG |
| CDK1-R | GCCACACTTCGTTGTTAGGA |
| CDK2-F | CTGGTCTGTTCATCGTGGTTCA |
| CDK2-R | CCTGGCTCATTGGTGGTACATT |
| CDK4-F | ATGTGGAGCGTTGGCTGTATC |
| CDK4-R | AGTCGTCTTCTGGAGGCAATC |
| CDK5-F | CTCTCTTCCCTGGCAATGATGT |
| CDK5-R | CGTTCACCAAGGATGTTGTAGC |
| CDK6-F | CCCATCGAGAAGTTTGTGACAG |
| CDK6-R | GCAGGTGAGAGTTCAGGTTGT |
| CDK7-F | AGGATGTATGGTGTGGGAGTAG |
| CDK7-R | CTGCTCTTCAGTTGGTGTACC |
| mCherry-F | CCGACTACTTGAAGCTGTCCTT |
| mCherry-R | TTGTAGATGAACTCGCCGTCCT |
| GFP-F | TTCACCGACAAGATCATCCGCAG |
| GFP-R | TGTCCACCACGAAGCTGTAGTAG |
| CDK2 3’ UTR -F | GAGCTGTACAAGTAATGCCCTTCCCAAAGCCCTT |
| CDK2 3’ UTR -R | GCGGATCCTGGGTGTAGCAGTCAGCA |
| Oligo name | Sequence (5’ - 3’) |
| CDK2 3’ UTR -F2 | CCAAAAGGCCCAGGGACAAG |
| CDK2 3’ UTR -R2 | CCCTGGGCCTTTTGGTCACA |
| CDK2 3’ UTR -R3 | GCGGATCCTGGGTGTAGCAGACAGCA |
| CREBBP 3’ UTR -F | GAGCTGTACAAGTAATTAAGCCTAGATGGGACTGT |
| CREBBP 3’ UTR -R | CTTTAAACATCAGACTGCCC |
| CREBBP 3’ UTR -F2 | GAGCTGTACAAGTAATTAAGCCTAGATGGGTCTGTGTC |
| CREBBP 3’ UTR -R2 | CTTTAAACATCAGACTGCCC |
| CREBBP 3’ UTR -F3 | GTCTGATGTTTAAAGAAACAA |

**Table S2. m6A modification information of CDKs**

| Gene | Total m6A modifications | 3’UTR m6A modifications |
| --- | --- | --- |
| CDK1 | 4 | 1 |
| CDK2 | 4 | 3 |
| CDK4 | 0 | 0 |
| CDK5 | 3 | 0 |
| CDK6 | 2 | 1 |
| CDK7 | 6 | 0 |
| Cdc25a | 7 | 4 |

**Table S3. Information of potential MA2 targets**

| Targets of MA | Concentration of MA | Affected pathways |
| --- | --- | --- |
| AKR1C3 | 100 μM | Reduction of PGD2 |
| KCNQ2/Q3 | 12 μM | KCNQ2/Q3 K^+^ currents |
| 11βHSD | 100 μM | PG biosynthesis |
| Cox2 | 5.5 μM | PG biosynthesis |
